# Abdominal erythema ab igne—beyond the rash

**DOI:** 10.1007/s10067-024-06998-1

**Published:** 2024-05-10

**Authors:** Roland van Rensburg, Helmuth Reuter

**Affiliations:** 1 Department of Medicine, Faculty of Medicine and Health Sciences, Stellenbosch University, Cape Town, South Africa; 2 Mediclinic Winelands Institute of Orthopaedics and Rheumatology, Stellenbosch, South Africa

**Keywords:** Carcinoma, Erythema ab igne, Rash

**Fig. 1:**
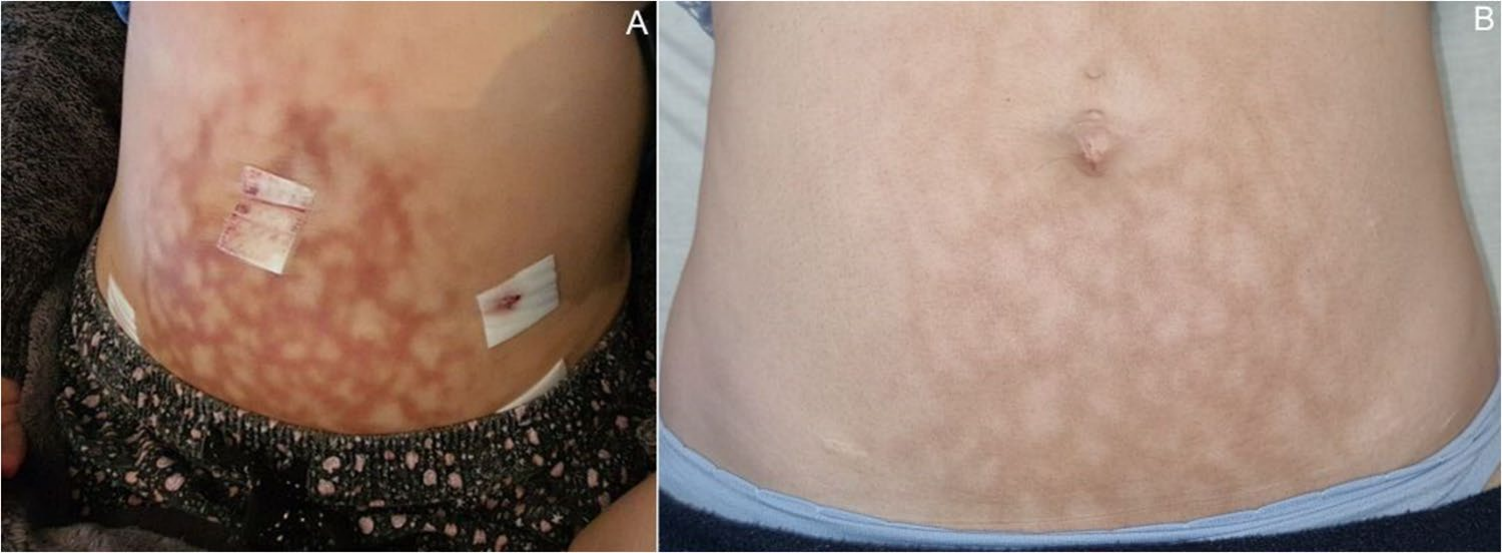
Abdominal erythema ab igne at the time of laparoscopic endometrial ablation (**A**). The hyperpigmentation at a follow-up visit 5 years later had improved, but was still present (**B**)

A 28-year-old female with Scheuermann disease presented to the rheumatology and chronic pain clinics for follow-up. She is known with fibromyalgia and hypermobility, presenting as widespread chronic pain and joint laxity, bladder pain syndrome, irritable bowel syndrome, and depression. She had a longstanding history of severe abdominal pain since menarche at 13 years of age, which was eventually diagnosed as endometriosis. The patient used hot water bottles extensively on her abdomen to alleviate the pain, resulting in striking abdominal erythema ab igne that was noted at the time of laparoscopic ablation of the endometriosis at 23 years of age (Fig. 1A). A sacral neuromodulator device was also implanted for the bladder pain syndrome, resulting in the abdominal pain improving significantly. She did not use hot water bottles since the combined management approach, but the characteristic reticular hyperpigmentation pattern—although improved—was permanent 5 years later at follow-up presentations (Fig. 1B). The patient was however concerned about further skin changes over the areas of discolouration, in particular the possibility of malignancy. She was counselled and referred to a multidisciplinary team for dermatological follow-up.

The hyperpigmentation of erythema ab igne is predominantly caused by epidermal atrophy and hemosiderin or melanin deposition due to infrared radiation from an external heat source [1]. With ongoing exposure, focal dyskeratosis with squamous atypia may present, increasing malignant transformation risk [2]. As such, longstanding or permanent erythema ab igne, as in this case, predisposes to the development of squamous cell carcinoma over the areas of hyperpigmentation [2]. As these patients are often kept under the care of a rheumatologist for primary pain conditions, they should be counselled and followed up periodically to monitor for any skin changes over the area. This includes Marjolin ulcers, which are aggressive squamous cell carcinomas arising from previous scar tissue or chronically inflamed tissue [3]. Younger patients also appear to have an increased risk, as a Swedish registry study showed that the standardized incidence rate for squamous cell carcinoma in rheumatoid arthritis patients < 50 years old was 2.37 (95% confidence interval [CI] 1.46 to 3.62) compared to 1.89 (95% CI 1.68 to 2.12) for all age groups [4]. A vigilant index of suspicion should be maintained especially in younger rheumatological patients with an area of longstanding erythema ab igne, and if any skin changes are noted, biopsy and/or prompt dermatology referral is warranted.
